# Numerical Simulation of Failure Behavior of Brittle Heterogeneous Rock under Uniaxial Compression Test

**DOI:** 10.3390/ma15197035

**Published:** 2022-10-10

**Authors:** Jia Liu, Fengshan Ma, Jie Guo, Tongtong Zhou, Yewei Song, Fangrui Li

**Affiliations:** 1 Key Laboratory of Shale Gas and Geoengineering, Institute of Geology and Geophysics, Chinese Academy of Sciences, Beijing 100029, China; 2 Innovation Academy for Earth Science, Chinese Academy of Sciences, Beijing 100029, China; 3 University of Chinese Academy of Sciences, Beijing 100049, China

**Keywords:** heterogeneous rock, Weibull distribution, optimal combination of micro-parameters, failure mode, heterogeneous specimen with discontinuities

## Abstract

Rocks have formed heterogeneous characteristics after experiencing complex natural geological processes. Studying the heterogeneity of rocks is significant for rock mechanics. In this study, a linear parallel bond model with Weibull distribution in two-dimensional particle flow code (PFC2D) is adopted to study the mechanical characteristics and brittle failure mode of granite rock specimens with different heterogeneity. Firstly, we selected several combinations of key micro-parameters of the parallel bond model. Then, we subjected them to a Weibull distribution to satisfy heterogeneity, respectively. Finally, we chose one optimal combination plan after comparing the stress–strain curves of heterogeneous rock specimens. We analyzed the simulated results of heterogeneous rock specimens. The crack distribution of rock specimens under peak stress shows different characteristics: a diagonal shape in rock specimens with low heterogeneity indexes, or a rotated “y” shape in rock specimens with high heterogeneity indexes. As for failure mode, the numerical simulation results show high consistency with the laboratory experiment results. The rock specimen breaks down almost diagonally, and the whole specimen tends to form an x-shaped conjugate shear failure or the well-known “hour-glass” failure mode. With the increase of the homogeneity index of the rock specimen, the shear rupture angle becomes larger and larger. Generally, the crack number increases with time, and when the rock specimen reaches the peak failure point, the number of cracks increases sharply. The development of cracks in numerical rock specimens under compression test is a result of the coalescence of many microscopic cracks. Furthermore, tensile cracks formed initially, followed by shear behavior along the macroscopic crack plane. We also preliminarily study the mechanical characteristics of heterogeneous rock specimens with discontinuous structural planes. The discontinuous structural planes are simulated by the smooth-joint model. We can conclude that the discontinuous structural planes and the microscopic structural planes which contribute to the heterogeneity have a mutual influence on each other.

## 1. Introduction

Rock is the aggregate of various minerals in nature and the product of a natural geological process, which has experienced a long geological time. Consequently, there are structural planes of various sizes in the rock mass, and there exist some microstructural planes in rocks, such as mineral cleavage, micro-cracks, inter-granular voids, lattice defects, and lattice boundaries, and other internal defects [1,2]. Microstructural planes in rocks are also called defects, and they can influence the physical properties and mechanical properties of rock mass [3,4,5,6]. In the early stages of rock mechanics, rocks were considered homogeneous and isotropic in some analytical models. These models typically simplify the physical properties or the force of the rock for analysis purposes [7]. However, discontinuities exist widely in natural rock masses [8], and the intact rocks are separated by different types of structural planes. In other words, the rock masses have different heterogeneity because of the macroscopic structural planes and microscopic structural planes in the rock mass. Heterogeneity becomes an important factor affecting material behavior [7]. This makes it imperative to study the heterogeneity and the structural plane of rock.

Numerous numerical investigations and experimental investigations have been conducted on rocks with microscopic structural planes and macroscopic structural planes [2,4,6,9]. Moreover, advanced techniques offer us chances to attain a more practical and even real-time development of microstructure, e.g., acoustic emission (AE) detection [10,11], the scanning electron microscope (SEM) [12], X-ray micro-computed tomography (CT) [13,14]. As for crack propagation, many scholars have conducted laboratory experiments with natural crack rock and rock-like materials [5,8,15,16]. At present, the widely used numerical simulation methods in geomechanics can be divided into continuum analysis and non-continuum analysis. The former mainly includes the finite element method [17,18], the boundary element method [19,20], the finite difference method [21], etc.; the latter mainly includes the block discrete element method [22], the particle discrete element method [16], the discontinuous deformation analysis [23], etc. Overall, laboratory experiments are usually interfered with by many factors, time-consuming and laborious. In addition, those non-destructive, advanced techniques are expensive, while the numerical simulation method, as a useful supplement, has many advantages: the numerical simulation method is efficient, cost-effective, repeatable, and the transient states of fracture development can be captured [7,8].

As a powerful discrete element method, the particle flow code (PFC) has been widely applied to many geotechnical problems [9,24,25,26,27], and the simulation results are in good agreement with the realistic situation. Furthermore, statistical modeling has become a popular technique applied in geomechanics [19]. For instance, the Weibull distribution is widely used in the study of rock heterogeneity [7,28,29,30]. Although experimental and numerical studies are numerous, studies on the selection of parameters that follow the Weibull distribution are rare, as are studies considering the detailed fracture development process of rock specimens under uniaxial compression test. In this paper, we tried to contribute our effort in this aspect. We adopted a linear parallel bond model to construct a brittle granite specimen and selected optimal micro-parameters which followed a Weibull distribution to satisfy the heterogeneity. Furthermore, we apply this method to a larger-scale fractured rock specimen to study its deformation and failure characteristics under a uniaxial compression test.

## 2. Materials and Methods

### 2.1. Brief Description of PFC2D

**Figure 1:**
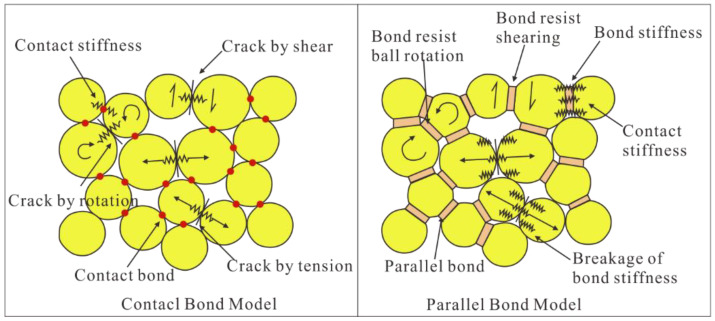
Illustration of two basic bonded-particle models (adapted from [35]).

The two-dimensional particle flow code (PFC2D) is a discrete element program in which the solid materials are represented as the assembly of circular particles. The model is populated with bodies, pieces and contacts, particle (ball) is one of three types of bodies. The motion of particles follows Newton’s second law: F = ma (”F” represents force, ”m” represents mass, ”a” represents acceleration). The particles are bonded together at the contact points [31]. The bond contact will be broken when the local contact force reaches the critical values. There are many kinds of embedded contact models in the commercial software PFC2D (Particle Flow code), which includes “null”, “linear”, “linear contact bond”, “linear parallel bond”, “hertz”, “hysteretic”, “smooth joint”, “flat joint”, etc. Two basic bonded-particle models (BPMs) of them are available, namely the ‘‘contact bond model” and the ‘‘parallel bond model [31,32,33]. The major advantages of BPMs are that complex empirical constitutive behavior can be replaced by simple particle contact logic [5], which can be utilized to simulate the mechanical behavior of rock very well. For more details, please refer to Yang [26]. The parallel bond model (PBM) has been widely used in previous research due to the advantage that the parallel bonds can transmit force and moment between the pieces. The force and moment can be related to the maximum normal stress and the shear stress acting within the bond material at the bond periphery [26,34]. The schematic diagram is shown in Figure 1 [35].

### 2.2. Calibration of Micro-Parameters

**Figure 2:**
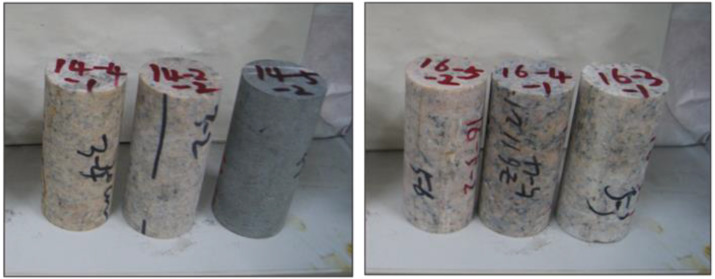
Pictures of rock specimens from laboratory experiments.

**Figure 3:**
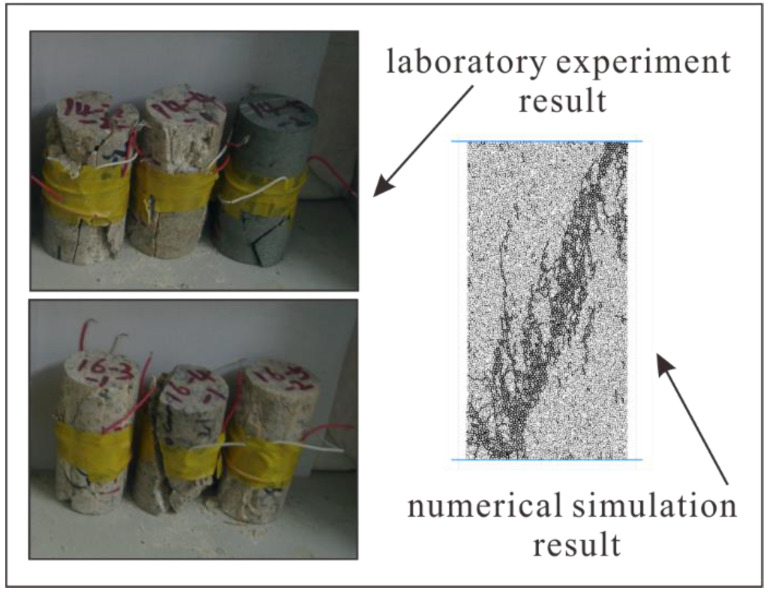
The comparison of laboratory experiment and numerical simulation results.

Although the uniaxial compression test is one of the simplest strength measures, it can give an indication of rock behavior under more complex stress systems [7]. In this article, uniaxial compression tests were applied onto several granite cylinders of 50 mm in diameter and 100 mm in height, which was collected from the Sanshandao gold mine, a Chinese coastal mine. The granite specimens are shown in Figure 2. We obtained the basic mechanical properties of the granite specimens, including the uniaxial compressive strength (UCS), the elastic modulus, and the Poisson’s ratio. As for numerical simulation, there are several critical parameters that have great influences on the mechanical properties of the rock specimen, e.g., the Young’s modulus of the particle, $E_{c}$, the ratio of normal to shear stiffness of the particle, $k_{n}/k_{s}$, the Young’s modulus of the parallel bond, ${\bar{E}}_{c}$, the ratio of normal to shear stiffness of the parallel bond,$\;{\bar{k}}_{n}/{\bar{k}}_{s}$, the particle radius, etc. The key preliminary work in PFC numerical simulation is to use the stress–strain curves of realistic granite specimens to calibrate the micro-parameters in the modeling. In other words, we attempt to obtain the same macroscopic mechanical properties with the trial and error method as those obtained from realistic laboratory experiments. Numerous previous works were focused on how to efficiently calibrate the micro-parameters [36,37,38,39]. The micro-parameters used in the PFC2D model for the granite specimen in this research are shown in Table 1. In addition, a comparison between the experimental mechanical properties and numerical mechanical properties of granite specimens is presented in Table 2. In the numerical simulation of the uniaxial compression test, the loading rate of the lower and upper rigid wall is set as 0.05 m/s. The time-step length “Δt” in this simulation is set as 3 × 10^−8^ s. Therefore, the rigid walls move vertically at a rate of 1.5 × 10^−9^ m/step, which is sufficient to ensure that the model runs in a quasi-static equilibrium state [40]. The comparison of laboratory experiment and numerical simulation results is shown in Figure 3.

### 2.3. Achieving Heterogeneity with Weibull Distribution

**Figure 4:**
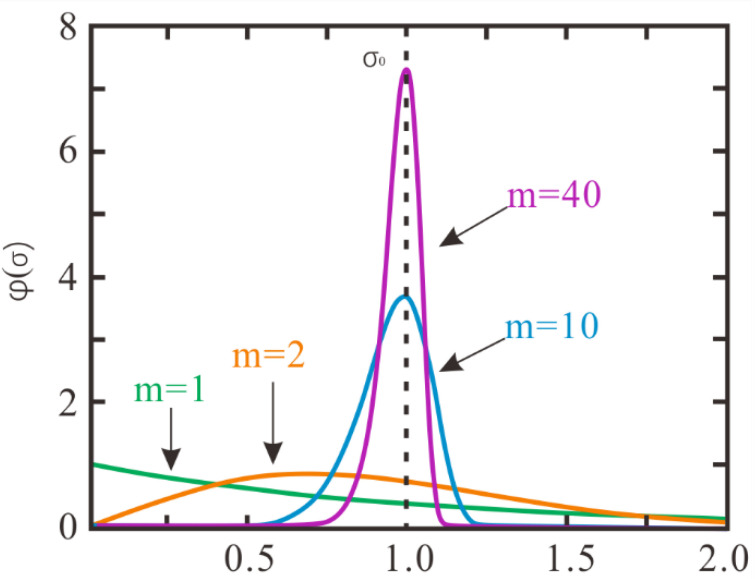
Four typical probability density curves of Weibull distribution for various homogeneity indices.

**Figure 5:**
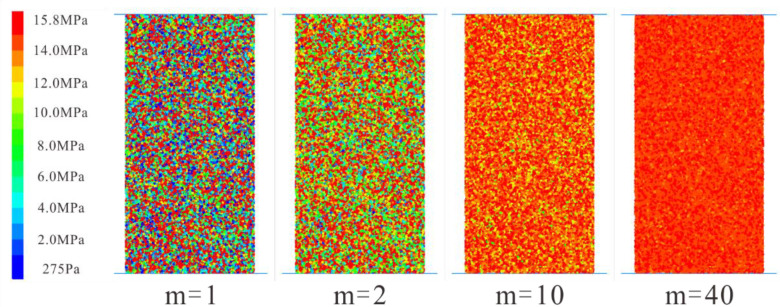
Four typical specimens with different homogeneity indices (take the “${\bar{\sigma }}_{c}$” parameter for example).

According to the preceding paragraphs, rocks in nature are heterogeneous materials, and the heterogeneity is a non-negligible property of rock deformation and failure. To study the heterogeneity, Weibull distribution n is introduced into numerical modeling to realize the randomness and heterogeneity of strength of rock specimen [7,41]. As for Weibull distribution, a probabilistic statistical method, the formula is shown below:

$$\varphi =\frac{m}{\sigma_{0}}{\left(\frac{\sigma }{\sigma_{0}}\right)}^{m-1}\exp\left[-{\left(\frac{\sigma }{\sigma_{0}}\right)}^{m}\right]$$

where σ is the micro-parameter value that follows the Weibull distribution; $\sigma_{0}$ is the reference value of the Weibull distribution of the micro-parameter value; m denotes the homogeneity index. The larger the value of m, the lower the heterogeneity of the rock specimen. In other words, the specimen with a higher m value is more homogeneous. To study the influence of rock heterogeneity on the uniaxial compression test, numerical specimens with different homogeneity indices (m equals 1, 2, 3, 4, 5, 10, 20, 30, 40) are generated, respectively. Four typical probability density curves of Weibull distribution for various homogeneity indices are shown in Figure 4. In addition, four typical specimens with different homogeneity indexes are illustrated in Figure 5 (take the “${\bar{\sigma }}_{c}$” parameter for example).

## 3. The Optimal Combination of Micro-Parameters

### 3.1. Build Different Micro-Parameter Combinations

In the previous chapters, we set the micro-parameters of numerical simulation which are consistent with the realistic rock specimens through the parameter calibration process. For heterogeneity, the micro-parameters in PFC2D simulation are randomly distributed by following a Weibull distribution. Tang has applied the Weibull distribution to the material properties of elements, which includes failure strength, $\sigma_{c}$, and elastic modulus, $E_{c}$ [7]. However, it’s worth noting that the micro-parameters in numerical simulation have nothing to do with the macro-mechanical properties before calibration. Therefore, we should pay more attention to selecting the micro-parameters which follow a Weibull distribution in PFC2D simulation. Relevant studies on selecting optimal micro-parameters are scarce and inadequate [30,40,42].

In this paper, we selected several combinations of key micro-parameters of the parallel-bond model. And subject them to a Weibull distribution to satisfy heterogeneity, respectively. The combination includes the following: (1) $E_{c}$ and ${\bar{E}}_{c}$ (use the abbreviation “d” to refer to this combination); (2) $\bar{c}$ (use the abbreviation “c” to refer to this combination); (3) ${\bar{\sigma }}_{c}$ (use the abbreviation “t” to refer to this combination); (4) $k_{n}$/$k_{s}$ and ${\bar{k}}_{n}$/${\bar{k}}_{s}$ (use the abbreviation “k” to refer to this combination); (5) $E_{c}$, ${\bar{E}}_{c}$ and $\bar{c}$ (use the abbreviation “dc” to refer to this combination); (6) $E_{c}$, ${\bar{E}}_{c}$ and ${\bar{\sigma }}_{c}$ (use the abbreviation “dt” to refer to this combination); (7) $E_{c}$, ${\bar{E}}_{c}$, ${\bar{\sigma }}_{c}$ and $\bar{c}$ (use the abbreviation “dtc” to refer to this combination).

### 3.2. The Uniaxial Compressive Test Results

**Figure 6:**
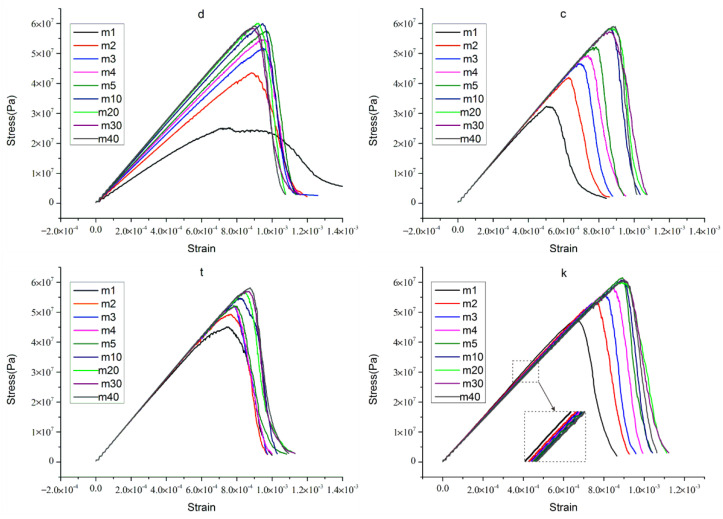
The stress-strain curves for heterogeneous rock specimens with micro parameters combination “d”, “c”, “t”, and “k”.

The axial stress–strain curves of heterogeneous rock specimens with micro-parameters combination “d”, “c”, “t”, and “k” are given in Figure 6. According to the peak value of curve and curve slope, we can obtain some information about the peak failure strength of rock specimen, the elastic modulus of rock specimen, and the deformation characteristics of rock specimen in the post-peak stage. (1) As for micro-parameter combination “d”, the peak failure strength of rock specimen increases with the increase of homogeneity index, except in cases where the homogeneity index equals 30 or 40. However, there is little difference in peak failure strength between rock specimens with a higher homogeneity index. The elastic modulus of rock specimen increases with the increase of the homogeneity index. When the rock specimens have low homogeneity indices (for example, m equals 1), the stress–strain curve shows obvious plastic characteristics, and the overall stress–strain curve demonstrates strain-softening behavior which was reported by previous articles [7,29,30]. (2) As for micro-parameter combination “c”: the peak failure strength of rock specimen increases with the increase of homogeneity index except in cases where the homogeneity index equals 30. Besides, the elastic modulus has little change with the increase of rock specimen homogeneity index. As for rock specimens with a low homogeneity index (m equals 1), the stress–strain curve also demonstrates strain-softening behavior. (3) As for micro-parameter combination “t”, the peak failure strength of rock specimen increases with the increase of homogeneity index. Nevertheless, the stress–strain curves of the rock specimens show unclear regulation in the post-peak stage. (4) As for micro-parameter combination “k”, the peak failure strength of rock specimen increases with the increase of homogeneity index. However, it’s interesting to find that the elastic modulus of heterogeneous rock specimens increases with the decrease of the homogeneity index. Similar simulation results can also be found in previous research [43,44,45]. This phenomenon shows that the effective stiffness ratio value of the whole rock specimen increases with the increase of the index of homogeneity. Therefore, the elastic modulus of the rock specimen decreases.

**Figure 7:**
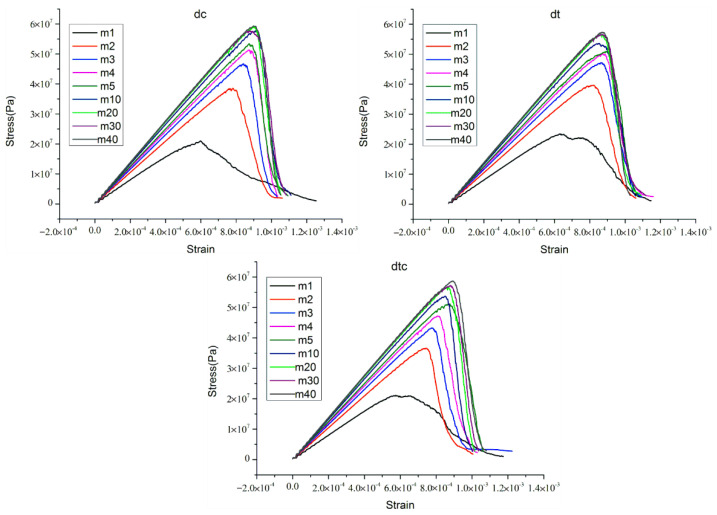
The stress−strain curves for heterogeneous rock specimens with micro parameters combination “dc”, “dt”, and “dtc”.

According to the experience of the preceding analysis, we analyze the following several combination cases ((5), (6), (7)). The stress–strain curves are displayed in Figure 7. As for micro-parameter combinations “dc”, “dt”, and “dtc”, the stress–strain curves for heterogeneous rock specimens have a lot in common. The peak failure strength of the rock specimen increases with the increase of homogeneity index, and so does the elastic modulus. The rock specimens with a low homogeneity index show plastic characteristics and strain-softening behavior in three situations. In addition, the rock specimens with high homogeneity index show obvious brittleness, and the strength loss is sharper in the post-peak stage. This phenomenon was documented by previous works [7,29,30,46]. Overall, the stress–strain curves with these three micro-parameter combinations have consistent characteristics.

## 4. Numerical Simulation Result of Uniaxial Compression Test

### 4.1. Stress-Strain Curve Results

According to the preceding analysis, the stress–strain curves of the last three micro-parameters combinations have consistent characteristics that accord with the previous works. Technically, three micro-parameters combinations all have better practical effects. However, we selected one of them (“dtc”.) to proceed the next analysis work considering previous works on calibrating micro-parameters [36,37,47]. Furthermore, the “dtc” stands for a situation where the $E_{c}$, ${\bar{E}}_{c}$, ${\bar{\sigma }}_{c}$, and $\bar{c}$ of the parallel-bond model are all subjected to the Weibull distribution. Figure 7 shows that the elastic modulus and the peak failure strength of rock specimen increase with the increase of homogeneity index. To make it easier to read, we draw a figure regarding the relationship between the homogeneity index and the peak failure strength, and the relationship between elastic modulus and homogeneity index in later sections of the paper. We will analyze the results later.

### 4.2. Failure Development of Heterogeneous Rock Specimen

#### 4.2.1. The Crack Distribution of Specimen under Peak Stress

**Figure 8:**
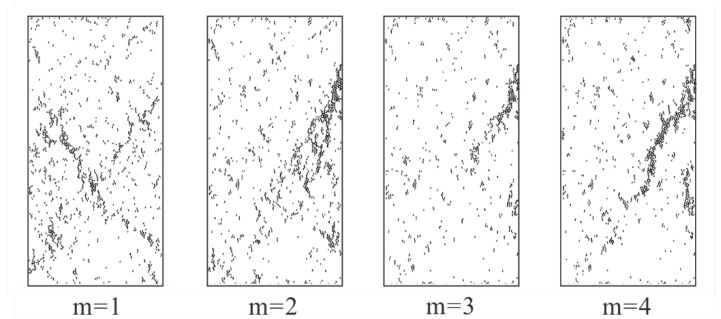
The crack distribution in rock specimens with low homogeneity indices (m equals 1, 2, 3, and 4) at peak failure strength.

We conducted uniaxial compression tests on rock specimens with different homogeneity index. In general, rock failure will be drastic in real laboratory experiments, it is impossible to capture the detailed characteristics of rock specimen in laboratory experiments. However, thanks to the advantage of PFC2D, we can easily obtain the failure state of the rock specimen during loading. As displayed in Figure 8, we collected the crack distribution of rock specimen with low homogeneity index (m equals 1, 2, 3, and 4) at peak failure strength. While the homogeneity index is low (m equals 1), the cracks exhibit a x-conjugate shape in the lower middle of rock specimen when the stress of rock specimen reaches its peak. As for other rock specimens with low homogeneity indices (m equals 2, 3, and 4), the cracks appear to be clustered along a lower diagonal (from the bottom left to the top right) when the stress reaches its peak. Overall, the cracks distribution becomes more concentrated with the increase of homogeneity indices.

**Figure 9:**
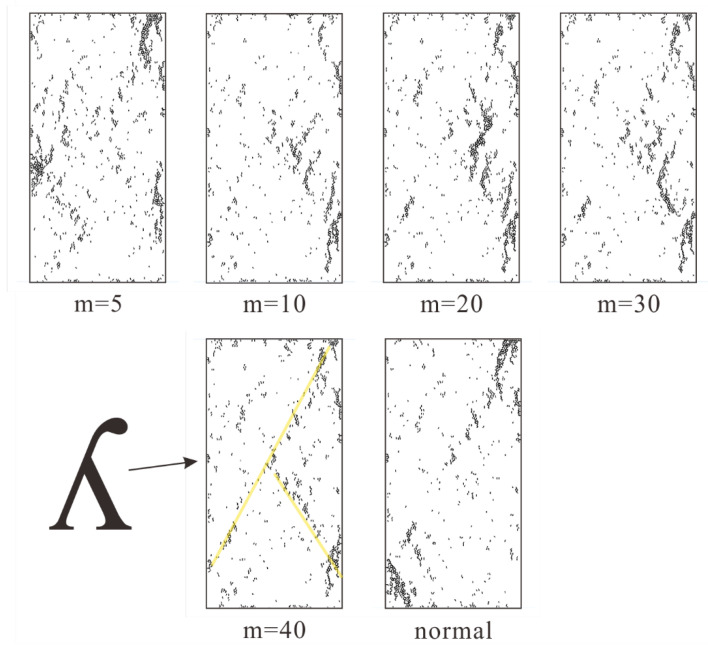
The crack distribution of rock specimen with low homogeneity index (m equals 5, 10, 20, 30, 40 and completely homogeneous normal condition) at peak failure strength.

As displayed in Figure 9, we collected the crack distribution of rock specimens with low homogeneity indices (m equals 5, 10, 20, 30, 40, and completely homogeneous normal condition) at peak failure strength. As for heterogeneous rock specimen (m equals 5), the crack distribution is relatively discrete and exhibits poor regularity. This type can be regarded as a transitional type. As for other rock specimens, when the stress reaches its peak, the main cracks appear to be clustered along a lower diagonal (from the bottom right to top left), and a small number of cracks gradually developed along the diagonal (from the bottom left to the top right) with the increase of homogeneity index. In other words, the shape of the clustered cracks looks like a “y” which rotates 180 degrees clockwise. The “y” becomes clearer with the increase of homogeneity index. In addition, the crack distribution pattern of a heterogeneous specimen is increasingly similar to the completely homogeneous, normal specimen.

#### 4.2.2. The Failure Mode

**Figure 10:**
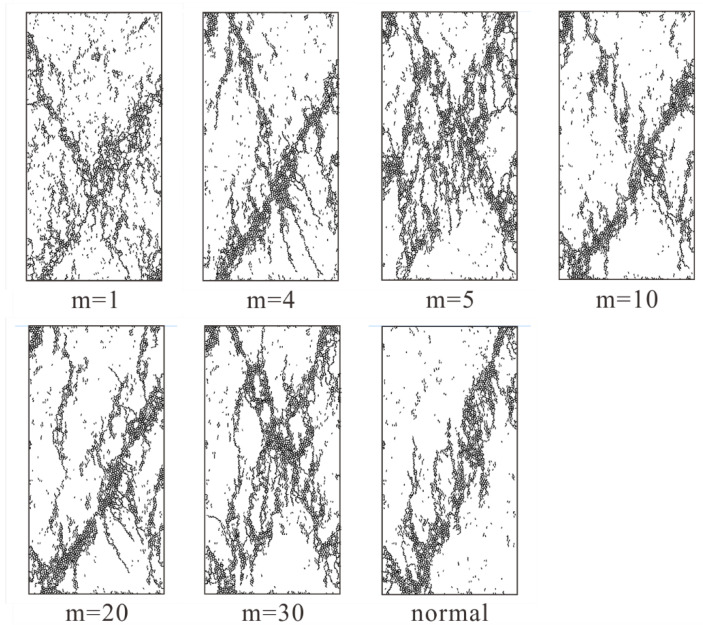
Several typical crack distribution results.

We all know that after the rock specimen reaches its peak strength, its internal structure has already been destroyed. However, the specimen basically keeps the original whole shape. Next, the cracks develop rapidly, cross and connected with each other to form the macroscopic fracture plane. Based on the preceding works, we can conclude that the crack distribution of specimen under peak stress is different. However, the final crack distribution pattern is similar when the macroscopic fracture plane of rock specimen forms. Figure 10 displays several typical crack distribution results. The shape of the clustered cracks looks like a “y” or a “x”, and some clustered cracks form a diagonal.

**Figure 11:**
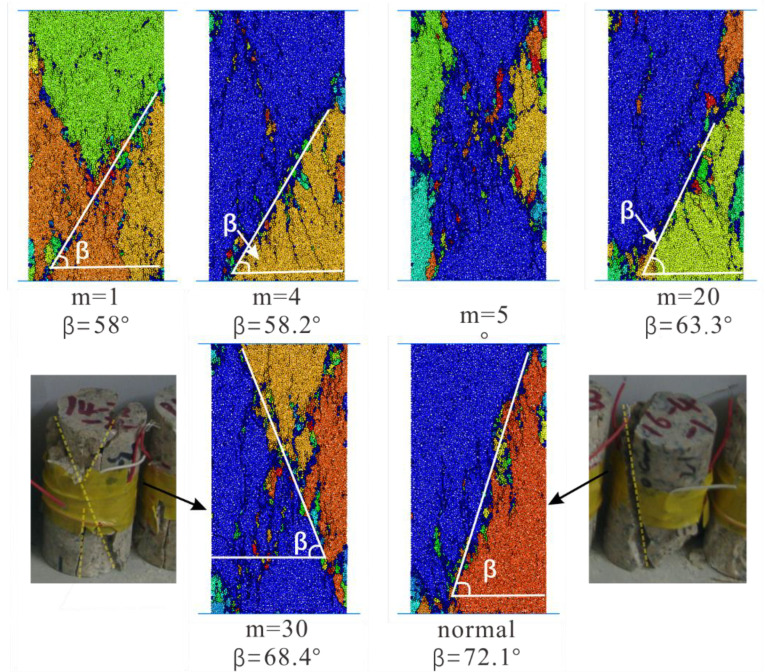
The broken rock specimens after the laboratory experiments and the numerical simulation tests (Different colors indicate different segments).

As displayed in Figure 11, we present the broken rock specimens after the laboratory experiments and the numerical simulation tests. The numerical simulation results show high consistency with the results from laboratory experiments. The rock specimen breaks down almost diagonally. Another diagonal of specimen is also unstable, and the whole specimen has the tendency to form an x-shaped conjugate shear failure or the well-known “hour-glass” failure mode. These failure modes are typical in laboratory experiments. The rupture angle represented by “β” is the angle between the load axis and the normal line of the rock specimen failure plane, which is positively correlated with the internal friction angle of rock. This can be interpreted in the following text: According to the Coulomb criterion [48], the higher the internal friction angle of the rock, the higher the strength of the rock. Furthermore, the higher internal friction angle will cause a higher rupture angle [49,50,51]. With the increase of homogeneity index of rock specimen, the rupture angle becomes larger and larger. As for the heterogeneous rock specimen (m equals 5), we can find in Figure 11 that several macroscopic fracture planes are almost vertical, and the rupture angle is almost 90°. This is one of three typical failure modes of rock in uniaxial compression tests, which is a tensile failure. The rock specimen will generate transverse tensile stress under the action of axial compressive stress. According to Figure 11, we found a similar phenomenon of macroscopic crack planes filled with the debris of little broken beams as Tang [7], which has been noticed in laboratory experiments.

## 5. Discussion

### 5.1. The Forming and Evolution of Cracks

**Figure 12:**
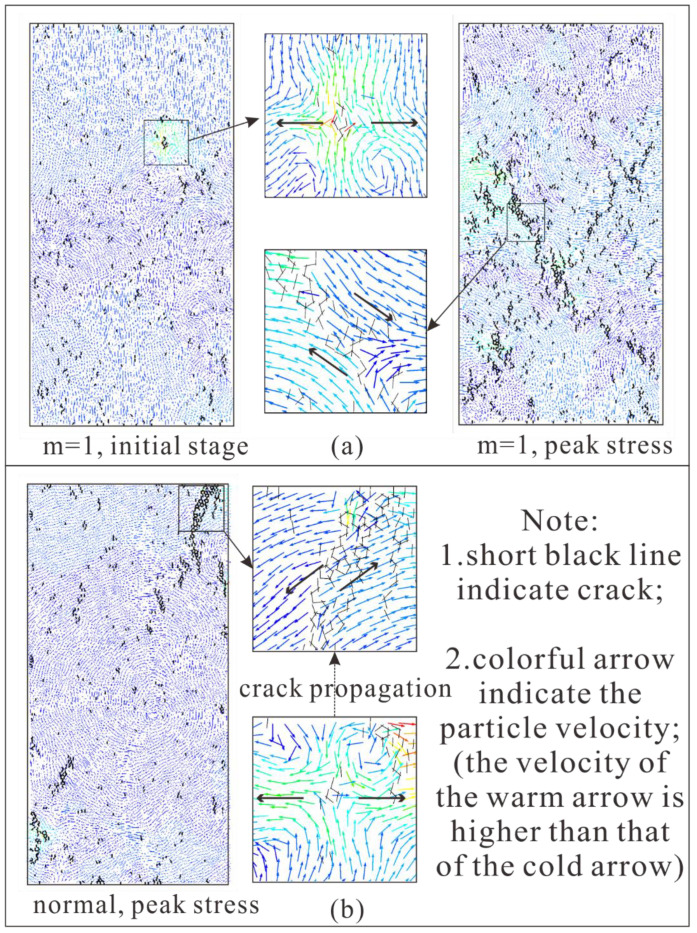
The development of cracks in numerical rock specimen (the black arrow indicates the direction of motion of particles). (**a**) The heterogeneous rock specimen; (**b**) The homogeneous rock specimen.

At first, numerous randomly distributed almost-vertical cracks develop in the rock specimen, they are regarded as tensile cracks (Figure 12). As pointed out by Tang [7], “It is known as axial splitting and cleavage at the macroscopic and the microscopic level, respectively”. In Figure 12, the black arrow indicates the direction of motion of particles. In Figure 12a, the small image in the upper middle shows the formation process of the tensile crack; the small image in the lower middle shows the formation process of the tensile crack. In initial stage, the heterogeneous rock specimen (m equals 1) develops some randomly distributed tensile cracks; and when it reaches peak stress, the cracks turn to shear cracks, propagate, and a macroscopic fracture plane gradually forms. We can learn the similar crack pattern of a completely homogeneous normal rock specimen from Figure 12b. The development of cracks in numerical rock specimens under compression test is a result of the coalescence of many microscopic cracks. Furthermore, tensile cracks are formed initially, followed by shear behavior along the macroscopic crack plane. Meanwhile, the rock specimen reaches peak stress. Henceforth, the deformation of rock specimen is mainly characterized by block slip along the macroscopic crack surface, and the bearing capacity of the rock specimen decreases rapidly with the severe deformation.

**Figure 13:**
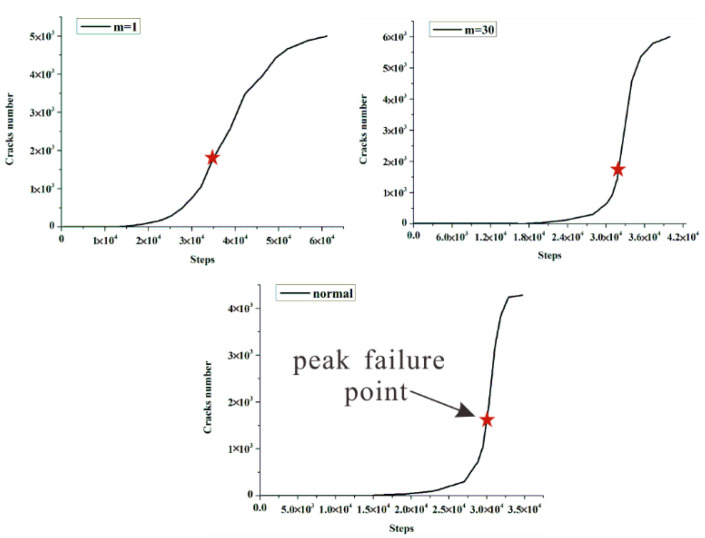
The cracks number increases over time.

As displayed in Figure 13, the crack number increases over time, and rises sharply when the rock specimen reaches the peak failure point. Moreover, the crack number still increases sharply in the post-peak stage until the specimen completely loses strength. Remarkably, the crack number of the heterogeneous rock specimen (m equals 1) does not show a drastic increase in the post-peak stage. On the contrary, the growth has slowed. In the same time, in combination with the corresponding curve (m equals 1) in Figure 7, the post-peak stress–strain curve gradually becomes gentler, and we can draw a deduction that this phenomenon may be related to the strain-softening behavior of the rock [7,52].

### 5.2. The Stress-Strain Curve

**Figure 14:**
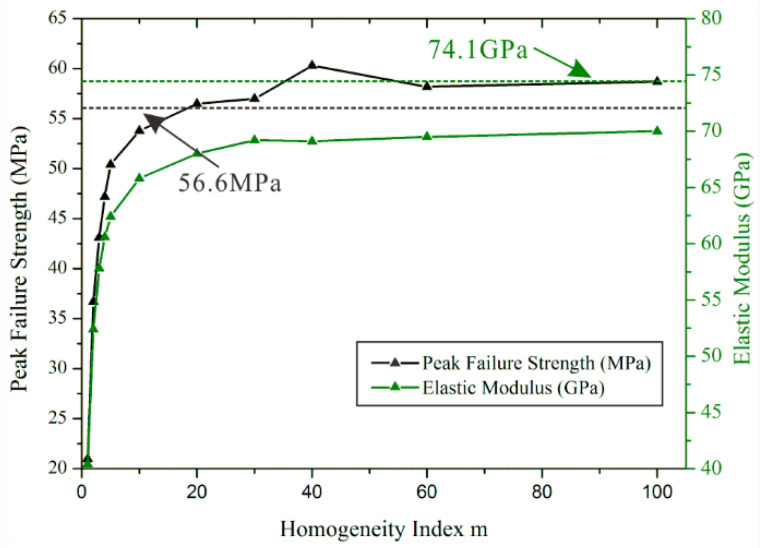
The peak failure strength values and elastic modulus values of heterogeneous and homogeneous rock specimens.

As shown in Figure 14, we displayed the elastic modulus values and the peak failure strength values by dealing with the stress–strain curves of heterogeneous rock specimens. At the same time, we indicate the peak failure strength (black dotted line) and elastic modulus (green dotted line) of homogeneous rock specimens respectively. We can find the interesting phenomenon that the gradually stable peak failure strength value is 4% bigger than the value of a homogeneous rock specimens. However, the gradually stable elastic modulus value is 5.5% smaller than the value of a homogeneous rock specimen.

Based on the previous work, we try to analyze this interesting phenomenon. It is well-known that the uniaxial compression strength may be regarded as the largest stress that a rock specimen can carry, which is much greater than the tensile strength of a rock specimen. Cracks will initiate, propagate and form coalescence when rock specimens are subjected to compression stress. In other words, the development of cracks is related to the stress on rock specimens. In heterogeneous rock specimens, the propagation and coalescence of cracks are not concentrated as normal homogeneous rock specimens. Consequently, the weak path is likely to form in heterogeneous rock specimens because of the increase of possible ways. Therefore, we believe that the smaller elastic modulus values are due to the effect of the relatively discrete cracks. Likewise, the relatively concentrated cracks in homogeneous rock specimens more easily form a macroscopic failure plane than the heterogeneous rock specimen. Therefore, the uniaxial compression strength will be affected.

### 5.3. Expand the Application

**Figure 15:**
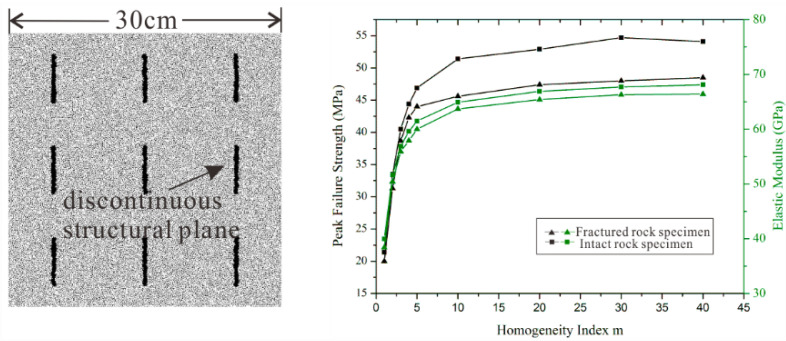
The mechanical properties of fracture rock specimen and intact rock specimen.

Rock mechanics is a science about the law of deformation and failure of deformed rock mass with environmental stress change. The rock mass is composed of rock and discontinuous structural planes. Apart from the mentioned microstructural planes in rocks (mineral cleavage, voids, lattice defects, internal defects, etc.), there exist some discontinuous structures on a large scale. Therefore, we try to study the mechanical properties of heterogeneous rock specimens with discontinuous structural planes. Due to the preliminary nature of the study, we only conducted the simulation of structural planes with simple distribution (Figure 15). The discontinuous structural planes are simulated by the smooth-joint model, and we also adopt the parallel bond model with the Weibull method to accomplish the heterogeneity of rock specimens. As displayed in Figure 15 (the dotted lines indicate the peak failure strength (black dotted line) and elastic modulus (green dotted line) of homogeneous rock specimens respectively), there is not much difference between the mechanical properties of fracture rock specimen and intact rock specimen when the rock specimens have a low homogeneity index (m equals 1, 2, 3, 4, and 5); and the difference between the mechanical properties of the fractured rock specimens and the intact rock specimen gradually keeps a stable value when the homogeneity is increasing. In other words, when the microscopic structural planes in rocks are numerous (the rock is too heterogeneous), the macroscopic discontinuous structural planes have little effect on rock failure. However, it works when the homogeneity index of rock specimens is increasing. Therefore, we can conclude that two structural planes have a mutual influence on each other. This may be a new way to study rock mass deformation. Thus, further studies in this area are necessary in our future works.

## 6. Conclusions

To examine the mechanical characteristics and failure behavior of brittle heterogeneous granite, numerical simulation by PFC2D software was performed for specimens with different heterogeneity. There are some key microscopic parameters in PFC2D, and we pay more attention to selecting the optimal micro-parameters that follow a Weibull distribution in numerical simulation. We selected several combinations of key micro-parameters of the parallel-bond model and we subjected them to a Weibull distribution to satisfy heterogeneity, respectively. Finally, we selected the “dtc” ($E_{c}$, ${\bar{E}}_{c}$, ${\bar{\sigma }}_{c}$ and $\bar{c}$) combination type. The peak failure strength of rock specimens increases with the increase of homogeneity index, and so does the elastic modulus. The rock specimens with low homogeneity indices show plastic characteristics and strain-softening behavior. In addition, the rock specimens with high homogeneity indices show obvious brittleness, and the strength loss is sharper in the post-peak stage. Simulation results show that the combination method is effective.

According to the simulated results, the two mechanical properties (the peak failure strength and the elastic modulus) of rock specimen both tend to keep a stable constant value with the increase in homogeneity. Besides, the crack evolution characteristics and the failure modes are developed. Overall, the cracks distribution becomes more concentrated with the increase of homogeneity index. The shape of the clustered cracks looks like a “y” which rotates 180 degrees clockwise. The “y” becomes clearer with the increase of homogeneity index.

As for failure mode, the results of the numerical simulations show high consistency with the results from laboratory experiments. The rock specimen breaks down almost diagonally. Another diagonal of specimen is also unstable, and the whole specimen has the tendency of forming an x-shaped conjugate shear failure or the well-known “hour-glass” failure mode. Our simulation results proved that the heterogeneous rock specimen (m equals 1) develops some randomly distributed tensile cracks in the initial stage; and when it reaches peak stress, the cracks turn to shear cracks, propagate, and a macroscopic fracture plane gradually develops. In a word, tensile cracks are formed initially, followed by shear behavior along the macroscopic crack plane. The crack number increases over time, and rises sharply when the rock specimen reaches the peak strength.

Furthermore, we simulated the discontinuous structural planes using the smooth-joint model. We also adopt the parallel bond model with the Weibull method to accomplish the heterogeneity of rock specimens. We can conclude that the discontinuous structural planes and the microscopic structural planes which contribute to the heterogeneity have a mutual influence on each other. When the microscopic structural planes in rocks are numerous (the rock is too heterogeneous), the macroscopic discontinuous structural planes have little effect on rock failure. However, it works when the homogeneity index of rock specimens is increasing.

## Figures and Tables

**Table 1:** The micro-parameters used in the PFC2D model for granite specimens.

Micro-Parameters	Value
Minimum limit value of radius ($r_{min}$) (mm)	0.3
Ratio of upper and lower limits of radius ($r_{max}$/$r_{min}$)	1.67
Porosity, φ	0.1
Density	2580
Young’s modulus of the particle, $E_{c}$ (GPa)	36
Ratio of normal to shear stiffness of the particle, ${\bar{k}}_{n}$/${\bar{k}}_{s}$	1.5
Particle friction coefficient (μ)	0.58
Young’s modulus of the parallel bond, ${\bar{E}}_{c}$ (GPa)	36
Ratio of normal to shear stiffness of the parallel bond,$\;{\bar{k}}_{n}$/${\bar{k}}_{s}$	1.5
Parallel-bond tensile strength (${\bar{\sigma }}_{c}$), (MPa)	15
Parallel-bond cohension ($\bar{c}$), (MPa)	28
Parallel-bond friction angle ($\bar{\Phi }$) (°)	30

**Table 2:** The comparison between the numerical and experimental mechanical properties of granite specimen.

	Laboratory Experiment Results	Numerical Simulation Results
Density, ρ (g/cm^3^)	2663	2580
Yong’s modulus, E (GPa)	77.56	74.1
Uniaxial compressive strength, UCS (MPa)	56.74	56.6
Poisson’s ratio, ν	0.2	0.2

## Data Availability

Not applicable
